# Recent Observations on Cancer of the Uterus

**Published:** 1901-01-26

**Authors:** 


### HOSPITAL CLINICS AND MEDICAL PROGRESS.

### RECENT OBSERVATIONS ON CANCER OF THE UTERUS.

DURING the last couple of weeks several papers have been published upon the subject of cancer of the uterus, a review of which will serve to focus attention upon this most distressing and fatal disease, and more especially upon what now seems to be the crux of the whole problem, so far as treatment is concerned, namely, early diagnosis.

### HÆMORRHAGE A SYMPTOM WHICH DEMANDS INVESTIGATION.

It is refreshing to find a physician who is not a specialist dealing with gynæcological affairs. Dr. Thomas Oliver,[1] lecturing on malignant diseases of the female genitalia, groups together a series of diseases which, although separated from each other by details of nomenclature and morphology, have in common one general characteristic, that of malignancy. It is characteristic of them all that they pass beyond the organ in which they originate, and invade other organs and tissues; that no tissue, not even the hardest, seems capable of arresting them; and that when removed by surgical operation they tend to recur. Of these diseases epitheliomata and carcinomata originate in epithelial surfaces, or in cells that line tubular glands; sarcomata arise in connective tissue; malignant adenomata in gland structure, like that of the uterus; while deciduoma malignum, taking its development from chorionic villi, is found in a uterus that has recently been emptied of its contents, for example, after pregnancy.

On the subject of diagnosis, and especially of early diagnosis—so essential if treatment is to be effective—Dr. Oliver lays especial stress upon the important indication given by any recurrence of bleeding from the uterus in women who have passed the climacteric, and who, having ceased menstruating for a year or more, have what they consider a return of their menses. This metrorrhagia is almost always due to malignant disease, and it is of the utmost consequence that one should not be led astray by the fact that on examination little or perhaps nothing may be discovered. One must not endeavour to explain away the hæmorrhage as being possibly due to some functional disturbance or to a hidden fibroid. The time for functional activity has in such a case passed by; nor is a fibroid which has long been quiescent likely to be forced into activity at such a time of life. If in a case in which this symptom occurs cancer is not obvious on ordinary examination, we must seek it; we must dilate the cervix and curette, and it is more than likely that some soft greyish-looking material will be removed which on microscopical examination will be found to be either sarcoma or malignant adenoma. In regard to treatment the cases may be divided into the operable and inoperable—practically into those that are discovered early and those that are discovered late. The cases in which the disease is limited to a very small part of the cervix, or, if in the uterus, where the womb is freely movable, with no indication in either instance of the disease having extended beyond the organ itself, are those suitable for operation, and, all things considered, even when the disease is limited to the exposed part of the cervix Dr. Oliver is disposed to recommend total extirpation as against amputation. In regard to the inoperable cases he has nothing favourable to say either for chian turpentine, Coley's fluid, arsenic, or thyroid extract.

### DIAGNOSIS OF CANCER OF THE BODY OF THE UTERUS.

Turning now to the specialists, one finds Dr. Handfield Jones,[1] in a lecture delivered at St. Mary's Hospital, dealing with the same question, but more especially directing attention to the points involved in the diagnosis of cancer of the body of the womb. He says that, familiar as we all are with the signs and symptoms of cancer of the cervix uteri, our knowledge is not so full or so accurate in regard to cases in which the disease commences in the body of the womb, and moreover that it is in the early stages of the mischief that our diagnosis is most often at fault; in other words, in the very stage when a correct diagnosis is the most important. In most cases when uterine hæmorrhage commences in a patient whose age is near fifty, and in whom the cervix is normal, we have to consider whether the bleeding is due (1) to some menopausal irregularity, or (2) to so-called senile endometritis, or (3) to commencing adenoma, or (4) to the onset of malignant degeneration. In regard to Class 1 the diagnosis is as a rule comparatively easy, the hot flushings and other nervous phenomena, conjoined with the absence of any enlargement of the womb, and aided also by the fact that the bleeding is more menorrhagic than metrorrhagic in character, rendering the diagnosis fairly certain. In Class 2 also, although the uterus is slightly enlarged, there is usually a tolerably characteristic clinical history. The difficulty comes in cases of adenoma and cases of malignant degeneration, and examples are given in which, after careful dilatation, curettement, and examination of the material removed, and after the pronouncement by an eminent pathologist that the scrapings in no way suggested a diagnosis of malignancy, still, in the end the disease turned out to be malignant. The outcome of this lecture is certainly to throw doubt on the trustworthiness of microscopic examination of the scrapings from a suspected uterus. "At the best," he says, "the microscope can only give confirmatory evidence, and is not to be trusted as an absolute means of diagnosis." One must then be guided principally by the clinical picture; and of all clinical phenomena steady increase in the size of a uterus, after the menopause, is the one that can most constantly be relied upon. Again, return of hæmorrhage after curetting in a woman who has passed the change of life is a sign of prime importance, whatever the microscopic appearances may be. Should hæmorrhage return after a free scraping of the uterus in a woman who has passed the change of life, and should the body of the uterus remain large, it will be the best to extirpate the organ. Dr. Handfield Jones concludes then: 1. That in cases of corporeal cancer there is a stage of benign adenoma. 2. That uterine scrapings are not perfectly reliable. 3. Clinical signs are more valuable than microscopial evidence. 4. The degree of malignancy varies much and the disease may run a very slow course. 5. Rapid increase in the size of the body of the womb is the most valuable sign in determining the need for extirpation of the whole organ.

### THE RESULTS OF OPERATION.

If now we inquire as to the results of operation we may refer to the paper read by Dr. Arthur Lewers[2] before the Royal Medical and Chirurgical Society a little time ago, in which he described the after-results in

B

forty consecutive cases of vaginal hysterectomy performed for cancer of the uterus. Up to the year 1893 he had for the most part treated cases of cancer of the cervix that appeared suitable for operation by the supra-vaginal amputation of the cervix, and had reserved total extirpation for cases of primary cancer of the body of the uterus. Since then, however, he has become more and more convinced that to perform the supra-vaginal amputation in such a way as to give the best chance of non-recurrence is in the majority of cases a more difficult matter than to perform vaginal hysterectomy, and he has lately only performed the supra-vaginal amputation of the cervix for very early cases of cancer of the vaginal portion in which there is a limited and superficial growth.

Of the forty cases of vaginal hysterectomy of which details are given by Dr. Lewers, in twelve there has been no recurrence (in one of these it is now more than a year since the operation, and in the remaining eleven the time since the operation varies from two to seven years); in eighteen cases there has been recurrence; four patients have died; in two the nature of the disease cannot be proved by examination of the specimens; in three no definite information could be obtained as to whether the disease recurred or not; and in one case the disease turned out to be primary tuberculous ulceration of the cervix. We see then that of the forty cases 27·5 per cent. remained free from recurrence for a period ranging from two to seven years, and analysing the cases in which recurrence took place we find that there were no instances of this having occurred later than during the fourth year after operation, "so that when a patient has passed through four complete years without recurrence there seems to be some probability that she may remain perfectly well."

We need hardly say that these results are very striking and very hopeful. Much is probably due to the care taken by Dr. Lewers in the selection of his cases, his practice having been to examine his patients under anæsthesia, and only to operate when the disease has appeared, so far as physical examination could decide, to be limited to the uterus. When there is evidence that the disease has spread beyond the anatomical limit of the uterus in any direction it is hopeless, Dr. Lewers thinks, to expect any permanent benefit from vaginal hysterectomy, even if it be possible to perform the operation. No difficulty arises, then, in deciding the question when, as is often the case, it is at once evident that the growth has spread beyond the uterus. In other cases, however, where on ordinary examination the mobility of the uterus appears to be unimpaired, decision is not so easy. The uterus may be freely movable, and yet the base of the bladder may be involved. Again, the mobility at an ordinary examination may seem normal, yet when an attempt is made to draw the cervix down to the vulva a check may be felt at one side or the other, in the region of the broad ligaments or of the utero-sacral ligaments, due to the extension of the malignant growth in the corresponding direction, an extension which can generally be best appreciated by examining per rectum while the cervix is being held down with a volsella. Cases of this kind are certainly unsuitable for the radical operation, says Dr. Lewers, for it is not justifiable to perform an operation like vaginal hysterectomy as a palliative operation, that is, to perform it where one feels certain that the patient has not even a chance of obtaining permanent benefit. On the other hand, one must not be too ready to reject cases as hopeless on the ground of the extent of the disease or the size of the growth alone, for there may be a very extensive growth, and one extruding largely from the cervix, and yet if the infiltration does not seem to have extended beyond the uterus operation is advisable. Again, we find Dr. Lewers, like every other clinical observer, insisting upon the necessity of early diagnosis, and especially upon the importance of remembering the gravity of bleeding after the menopause, or of bleeding occurring at an earlier time of life between the menstrual periods, this being a symptom which always demands full and careful investigation. Let us not be led astray by an appearance of good health, for patients suffering from cancer of the uterus may, and generally do for a relatively long period, look quite well.

[1] Brit Med. Journ., Jan. 19 [2] Lancet, Jan. 5th.

